# Effect of haemarthrosis on the rehabilitation of anterior cruciate ligament reconstruction- single bundle versus double bundle

**DOI:** 10.1186/1749-799X-8-5

**Published:** 2013-03-19

**Authors:** Vibhu Bahl, Ankit Goyal, Vineet Jain, Deepak Joshi, Deepak Chaudhary

**Affiliations:** 1 Sports Injury Centre, Safdarjang Hospital, Delhi, 110092, India

**Keywords:** Haemarthrosis, Drain, Double bundle ACL, Isokinetic, Muscle strength

## Abstract

**Background:**

Haemarthrosis and pain adversely affects the functional outcome of ACL reconstruction, especially in case of DB ACL reconstruction due to more extensive procedure. The purpose of the study was to evaluate the effect of haemarthrosis on the rehabilitation of DB ACL reconstruction versus SB ACL reconstruction.

**Methods:**

100 patients were divided into two groups, of SB ACL and DB ACL reconstruction consisting of 50 patients each. An intra-articular drain was put in every patient. The pain was evaluated till week 8 using VAS (Visual Analog Scale). The Functional outcomes were evaluated using the Isokinetic Dynamometer at 3 and 6 months in both the groups. Muscle bulk and Range of motion were also noted in each group.

**Results:**

The results showed that there was statistically significant difference between the drain amount (n = 60.3 ml in SB ACL group vs. n = 94.2 ml in the DB ACL group) and haemarthrosis (n = 0.7 in SB ACL vs n = 1.5 in DB ACL) at week 1 post-operatively. Also the pain outcome improved on SB ACL after day 3 (VAS, n = 1.8) as compared to the DB ACL group (VAS, n = 3.7). The isokinetic muscle strength was found to be statistically significantly (p value < 0.05) better in the SB ACL group in the quadriceps muscle (both concentric and eccentric) at the end of the 3rd month. In the SB ACL group the Quadriceps Concentric strength deficit was 22.32% as compared to 34.12% in the DB ACL group. Both the groups had comparable flexor muscle strength at end of 3rd month. Both the groups had comparable muscle strength after 6 months of post-operative rehabilitation in both quadriceps and Hamstring muscle group.

**Conclusion:**

We noted that rehabilitation of DB ACL reconstruction group lags behind that of SB ACL reconstruction during the first 3 months due to post-operative haemarthrosis & its effects, but show comparable results after 6 months. The muscle strength measured isokinetically and the muscle bulk were found to be greater in the SB ACL group initially after 3 months but was found to be similar after 6 months.

## Introduction

Several studies have evaluated the role of haemarthrosis and intra-articular drains in Anterior Cruciate Ligament (ACL) reconstructive procedures, with most of them documenting the fact that intra-articular drains are not necessary in them as it doesn’t affect functional outcome [1-3]. These studies had incorporated only Single Bundle ACL (SB ACL) reconstruction procedures. There has been no study comparing the blood loss in SB ACL versus Double Bundle (DB ACL) reconstruction and its effect on the functional outcome.

The present study was undertaken to assess the blood loss and incidence of haemarthrosis in SB ACL reconstruction vis-a-vis DB ACL reconstruction and its effect on functional outcome. This study also aimed to evaluate the role of other variables like notchplasty, duration of surgery and associated meniscal procedures contributing to incidence of increased blood loss post-operatively.

## Materials & methods

This was a prospective trial conducted between January 2010 and January 2011, in which 100 male patients who underwent either Single Bundle or Double Bundle ACL reconstruction and available for follow up for 6 months were included in the study. There were 50 patients in each group. Ethical approval was obtained from the Ethical Committee/Institutional Review Board of Safdarjang Hospital.

Exclusion criteria included significant pain preoperatively (VAS > 5), a large swelling preoperatively (grade 3 or 4 effusion), revision cases, and patients with a known risk of bleeding (bleeding diathesis or prescribed anticoagulation medication). Female patients were not taken up for the study since invariably they have smaller knees and would require SB ACL reconstruction and thus could induce a bias in the results.

All the patients were informed about their surgery and explained in detail regarding the procedure and post-operative rehabilitation protocol. All surgeries were performed by the senior author at least 4 weeks after trauma, when the patients had regained at least 120 degrees of ROM (range of motion), knee had become non-tender and effusion had subsided.

### Surgical technique

Spinal anaesthesia was given in all patients and a pneumatic tourniquet was used routinely. All patients received prophylactic intravenous antibiotics preoperatively (Ceftriaxone, 1gm).

Surgery was performed using standard 3 portal technique using Antero lateral (AL), Anteromedial (AM) and Accessory Anteromedial (AAM) portals. Semitendinosus and Gracilis tendon were harvested using a standard technique. The Semitendinosus (for the AM bundle) was doubled and gracilis tendon (for the PL Bundle) was tripled. The distal free ends of the tendon were stitched with Ethibond and the grafts were pretensioned. Either the soft tissue remnants of the ACL were identified on tibia and femur as a guide for the insertion sites of AM and PL bundles or the bony landmarks (lateral intercondylar ridge and lateral bifurcate ridge) were visualized to delineate the bundles attachment on the femoral side in chronic cases. Arthrex tibial ACL guide was used for making the tibial tunnels. The AM tibial tunnel was drilled first followed by PL tibial tunnel with a 2.4 mm drill bit. AM followed by PL tibial tunnel was reamed according to graft size. The AM femoral tunnel was drilled next using the trans-tibial approach. The depth of the femoral tunnel was kept at 35 mm. The PL femoral tunnel was drilled and reamed next using the accessory anteromedial (AAM) portal and visualizing the PL bundle footprint. The tunnel reamed up to 30 mm with a cannulated reamer which was matched to the graft diameter. This tunnel was extended to the lateral femoral cortex with the 4.0 mm cannulated reamer. The gracilis graft for the PL bundle is fixed at the femoral side using a Bioscrew. AM graft is then fixed on the femoral side using cross pin femoral instrumentation. The knee was then cycled several times through the range of motion and the PL graft is fixed at 0- 15 degree of knee flexion whereas AM graft is fixed at 45 degree of knee flexion using bioscrews of appropriate size.

Notchplasty was performed only if there was significant notch stenosis or when graft impingement was present. Full Range of Motion was confirmed intra-operatively and stability checked with Lachman Test. The intra-articular fluid was drained with a cannula after the procedure was over. An 18 G disposable suction drain was used in all cases and was put in an inside out method to confirm intra-articular placement of the drain. Compression dressing with ice-pack was applied before deflation of the tourniquet. Time taken for surgery, notchplasty and menisectomy if done were noted for each case.

All patients were put through a standardized rehabilitation protocol supervised by a physiotherapist with emphasis on achievement of early Range of Motion and weight bearing as tolerated. Axillary crutches were used till there was adequate quadriceps control and minimal limp. The drain was removed by a member of the study group on first post-operative day and drainage recorded.

The patients recorded their pain on a visual analogue scale (VAS) [4] at a consistent time in the evening on postoperative day 1, 2, and 3. They were reviewed in the clinic at 1, 4 weeks and then on monthly interval till 6 months. Pain (VAS), grade of haemarthrosis, ROM, thigh circumference and muscle strength as well as the presence of any complications was documented.

Primary outcome measures included grade of haemarthrosis, according to the classification of Coupens and Yates [3] (graded subjectively from 0 to 4) (Table 1) and pain measured by VAS. Secondary outcome measures included ROM, measured by use of a standard handheld goniometer, loss of muscle bulk and muscle strength recovery.

**Table 1:** Clinical grading of haemarthroses (Coupens and Yates)

**Grade**	**Description**
0	No detectable fluid
1	Fluid present with fluid wave
2	Palpable fluid in suprapatellar space
3	Ballotable patella
4	Tense haemarthroses

Thigh circumference was measured using an inch tape kept 2.5 cm above the superior pole of patella, evaluating the quadriceps muscle bulk in both the thighs and the difference calculated.

Muscle strength (Quadriceps and Hamstrings) was measured at the end of 12 weeks and 24 weeks with isokinetic muscle charting. Each subject underwent test to measure the isokinetic muscle strength (HUMAC/NORM Isokinetic muscle testing at 60/60 deg. /sec 3 Reps.) while performing flexion-extension movements with both involved and uninvolved knee joint. Isokinetic dynamometer measured two types of muscular contractions – eccentric isokinetic and concentric isokinetic contractions [5].

All the tests were evaluated statistically using unpaired t- tests and the p- value set at 0.05.

## Results

The amount of blood drained varied from a mean of 60.3 ml (range 40 ml to 120 ml) in SB ACL study group to a mean of 94.2 ml (range 50 ml to 150 ml) in the DB ACL group (Table 2). The subjective analysis of haemarthrosis, done after removal of drain, was statistically significant between the two groups till the end of fourth week. The mean value of the grade at the end of 4th week was 0.6 (range 0-1) in the SB ACL group compared to a mean of 1.1 (range 0-2) in the DB ACL group.

**Table 2:** Clinical comparison between SB ACL & DB ACL reconstruction group

	**SB ACL Group**	**DB ACL Group**	**p value**
**No.**	50	50	
**Age (yr)**	36 (Range 15-52)	28 (Range 21-38)	>0.05
**Time to surgery (weeks)**	12.2	13.1	>0.05
**Partial menisectomy**	31	36	>0.05
**Notchplasty**	12	23	<0.05
**Drain amount (ml)**	60.3 ml (Range 40 ml-120 ml)	94.2 ml (Range 50 ml-150 ml)	<0.05
**Tourniquet time (min.)**	42.1 min. (Range 34 min.-100 min.)	74.7 min.(range-50 min-150 min)	<0.05

The mean value of Visual Analog Scale (VAS) decreased from a mean of 2.7 at day 1 to 1.8 in the SB ACL group on day 3, while it came down from 4.2 to 3.7 in the DB ACL group. The differences in the recordings in both the groups remained statistically significant only till the end of 4th week (Table 3).

**Table 3:** Functional outcomes

		**Day 1**	**Day 2**	**Day 3**	**Week 1**	**Week 4**	**Week 8**
Pain	SB	2.7	2.2	1.8	1.6	1.2	0.5
VAS	DB	4.2	3.9	3.7	2.7	2.3	0.8
Range of motion	SB	NA	NA	NA	4.1°	3.2°	0°
Loss of terminal extension	DB	NA	NA	NA	8.3^o^	6.7^o^	1.2^o^
Loss of terminal flexion	SB	NA	NA	NA	60.2°	32 °	15°
	DB	NA	NA	NA	63.1^o^	34^o^	11^o^
*Thigh circumference deficit (cm)	SB	NA	NA	NA	2.2	0.8	0.3
	DB	NA	NA	NA	3.6	2.4	1.1
Haemarthrosis	SB	1.2	1.1	0.9	0.7	0.6	0
(Grade)	DB	2.3	2.2	1.8	1.5	1.1	0.3

*SB* = Single Bundle ACL group *DB* = Double bundle ACL group.

The other major outcome which was noted was the loss of Range of motion (ROM) calculated after the first week using the standard goniometer. There was statistically significant restriction of terminal extension at the end of first week in DB ACL group (p value < 0.05). The loss of extension was noted to be 4.1 degree in the SB ACL group as compared to 8.3 degree in the DB ACL group at the end of first week. The patients followed the same rehabilitation protocol and it was noted that the ROM were comparable at the end of 8th week in both the groups (Table 3). The loss of flexion was comparable in both the groups during the whole rehabilitation period.

The thigh circumference was measured at the end of week 1, 4 and 8. There was statistically significant (p value < 0.05) difference between the SB ACL group (n = 0.3 cm) as compared to the DB ACL group till 8 weeks (n = 1.1 cm) (Table 3).

The muscle strength testing was also done Isokinetically (measured by Torque in N/M^2^) at 12 and 24 weeks to evaluate the recovery of the muscle strength post ACL reconstruction. The quadriceps strength on concentric and eccentric contraction was found to lag behind in DB ACL group (p value < 0.05) at 3 months but was comparable to the SB ACL group at 6 months. The hamstrings (Concentric and Eccentric) power had statistically insignificant difference in (p value = 0.43) in both the groups even at end of 3 months (Table 4).

**Table 4:** Quadriceps and hamstring muscle strength comparison between SB ACL and DB ACL group

**Muscle testing**		**Normal knee**		**Operated knee**		**Deficit (%)**	
		**3 months**	**6 months**	**3 months**	**6 months**	**3 months**	**6 months**
Quadriceps (Concentric)	SB	38.26	40.36	29.73	35.24	22.32	12.7
	DB	39.32	42.34	25.91	32.66	34.12	16.8
Quadriceps (Eccentric)	SB	34.21	39.45	24.54	34.29	28.27	13.09
	DB	36.27	40.10	22.01	32.66	39.34	18.56
Hamstrings (Concentric)	SB	18.98	23.05	15.70	21.16	17.33	8.23
	DB	21.23	25.42	17.07	22.55	19.64	11.32
Hamstrings (Eccentric)	SB	15.1	19.63	13.08	18.35	13.4	6.56
	DB	18.46	22.31	15.72	20.31	14.88	9.80

*HUMAC/NORM Isokinetic muscle testing (torque = N/m^2^) at 60/60 deg/sec 3 Reps.

*In normal subjects, imbalances of 10% or less can be considered normal while differences of 10% to 20% possibly abnormal and those above 20% probably abnormal.

There were no infections in either group but 3 patients had to be aspirated (mean value-120 ml, range- 75 ml- 150 ml) in the DB ACL group at 3rd post-operative day for tense haemarthrosis whereas no patient in the SB ACL group had their knee aspirated.

There was no statistical difference in the number of menisectomies in both the groups. There was statistically significant difference in the number of patient requiring notchplasty and time taken for surgery, both being higher in the DB ACL reconstruction group (Table 2).

## Discussion

The Complications Committee of the Arthroscopy Association of North America formulated a registry to investigate complications associated with arthroscopy [6]. The overall complication rate in this study was 1.68%. Of the complications recorded, haemarthrosis occurred with a frequency of 60.1%. The actual significance of haemarthrosis has been debated [7]. Not only is there a toxic effect on the articular cartilage by the haemarthrosis but the susceptibility to infection is increased in its presence. In addition, it has been documented that in certain arthroscopic procedures the occurrence of haemarthrosis results in the scar formation, decreased range of motion, subsequent synovitis and delayed rehabilitation [8].

In our study the grade of the haemarthrosis and post – operative pain was significantly higher in the double bundle ACL group than the SB ACL group. This difference can be attributed to following factors associated with DB ACL reconstruction group i.e. increased surgical time (n = 74.7 min.), the numbers of tunnels drilled are doubled, in 46% of cases notchplasty was done due to graft impingement of the Antero Medial bundle (AM) on extension, this is especially true as more area is taken up in the intercondylar notch due to 2 grafts in the DB ACL reconstruction.

The significant difference in the range of motion between DB and SB ACL groups noted in terms of loss of terminal extension and difference in muscle bulk at the end of 4th week between the two groups can be attributed to the increased haemarthrosis and pain post – operatively leading to delayed rehabilitation of quadriceps.

Most of the studies which had compared difference in ROM and / or thigh circumference between DB ACL and SB ACL didn’t report significant difference on a long term follow-up, [9,10] but no study has compared the results in the initial 6 months of rehabilitation. McCormack et al [11] measured ROM and or thigh circumference in cases of SB ACL reconstruction only with and without drain and didn’t find any statistically significant difference at 1,4, 8 weeks.

The numbers of menisectomies done in both these groups were statistically similar and thus did not influence the result of our study.

Muscular strength was evaluated using the isokinetic machine at 12 and 24 weeks and there was a statistically significant difference in muscle strength deficit in the SB ACL group as compared to the DB ACL group. The SB ACL group had statistically significant better muscle strength at 3 months in the quadriceps muscle groups. This again can be attributed to pain and haemarthrosis post operatively in the DB ACL group delaying rehabilitation.

The results for quadriceps weakness can be explained due to relative inactivity and ineffective strengthening exercises following surgery which leads to type II muscle fibre atrophy [12-16]. Also the patients with ACL reconstruction demonstrate arthrogenic quadriceps inhibition in order to minimise anterior tibial translation and ACL graft strain [17]. During ACL reconstruction the gamma loop function could be attenuated in the quadriceps because the mechanoreceptors in the ACL are not surgically reconstructed [5,18]. In contrast to the quadriceps, the hamstrings are less susceptible to strength deficits following an ACL injury. These deficits are thought to be due to bi-arthrodial nature of three of the four hamstring components such that even when knee mobility is impaired following in ACL injury, hip extension continues to act as stimulus for the hamstrings [19-22]. Also the current rehabilitation protocols emphasis early and aggressive hamstring training following an ACL reconstruction [23-26] on the basis that hamstring contraction can produce posterior tibial translation to reduce the strain on the maturing ACL substitute [19]. While using semitendinosus and gracilis tendon autograft, recovery of hamstring strength is of some concern given that the semitendinosus tendon is sacrificed during the procedure [24] but most of the investigators have generally found non-significant hamstring deficits between the operated and non-operated site in the post-operative period [27-29].

## Conclusion

In our study we noted that the rehabilitation of DB ACL reconstruction group lags behind the SB ACL reconstruction during the first 3 months due to post-operative haemarthrosis and pain even after using drain but shows comparable results after 6 months. Further studies are needed comparing the results in DB ACL reconstructions, with or without intra-articular drain to further investigate these findings.

## Competing interests

The authors declare that they have no competing interests.

## Authors’ contribution

All the surgical procedures were performed by the senior surgeons DC and DJ, data collection including clinical & functional parameters in the pre-operative and post-operative period was done by AG. The study was designed and manuscript was drafted by VJ and data analysis was done by VB. All authors read and approved the manuscript.
